# Production and Characterisation of an Exopolysaccharide by *Bacillus amyloliquefaciens*: Biotechnological Applications

**DOI:** 10.3390/polym15061550

**Published:** 2023-03-21

**Authors:** Enrique Sánchez-León, Elisa Huang-Lin, Ricardo Amils, Concepción Abrusci

**Affiliations:** 1 Departamento de Biología Molecular, Facultad de Ciencias, Universidad Autónoma de Madrid, UAM, Cantoblanco, 28049 Madrid, Spain; 2 Centro de Biología Molecular Severo Ochoa, CSIC-UAM, 28049 Madrid, Spain

**Keywords:** exopolysaccharide, biodegradation, *Bacillus*, emulsifying, antioxidant

## Abstract

The *Bacillus amyloliquefaciens* RT7 strain was isolated from an extreme acidic environment and identified. The biodegradation capabilities of the strain using different carbon sources (glucose, oleic acid, Tween 80, PEG 200, and the combination of glucose–Tween 80) were evaluated via an indirect impedance technique. The glucose–Tween 80 combination was further studied using nuclear magnetic resonance (NMR). The exopolysaccharide (EPS_RT7_) that had been produced with the strain when biodegrading glucose–Tween 80 was isolated and characterised using different techniques (GC–MS, HPLC/MSMS, ATR–FTIR, TGA, and DSC), and its molecular weight was estimated. The results show that the average molecular weight of EPS_RT7_ was approximately 7.0794 × 10^4^ Da and a heteropolysaccharide composed of mannose, glucose, galactose, and xylose (molar ratio, 1:0.5:0.1:0.1) with good thermostability. EPS_RT7_ showed good emulsifying activity against different natural oils and hydrocarbons at high concentrations (2 mg/mL) and at the studied pH range (3.1–7.2). It also presented good emulsifying activity compared to that of commercial emulsifiers. Lastly, EPS_RT7_ showed antioxidant capacity for different free radicals, a lack of cytotoxicity, and antioxidant activity at the cellular level. EPS_RT7_ has promising applications in bioremediation processes and other industrial applications.

## 1. Introduction

The dispersion of large amounts of toxic polluting agents to the environment caused by natural or human activities leads to adverse impacts on population and ecosystem health [1]. Conventional remediation techniques such as the use of surfactants have received great attention when counteracting polluting activities [1].

Surfactants are compounds that reduce surface and interfacial tension at the interfaces of liquids, solids and gases in order to create emulsions with liquids [2]. These compounds are highly used in the industry in order to remediate contaminated sites from environmental pollutants such as hydrocarbons [3]. The most popular surfactants are synthetic chemical surfactants [4], which are generally toxic and lack biodegradability, leading to bioaccumulation [5]. Manufacturing these surfactants and their byproducts can adversely impact the environment. Surfactants can be disposed of in rivers or sewage treatment plants, which results in marine ecosystem pollution [6].

In order to replace these compounds, attention is given to compounds that are kinder to the natural environment, such as bioemulsifiers (BEs), biosurfactants (BSs) and exopolysaccharides (EPSs) [7]. EPSs produced by microorganisms are compounds with significant potential in various commercial applications such as the emulsification of various hydrophobic substrates, food, or the pharmaceutical industry [8,9]. They have significant advantages when it comes to biodegradability and effectiveness [10]. Within this category is a group of highly interesting polymers, such as exopolysaccharides from microorganisms (microbial exopolysaccharides (EPSs)). Their physicochemical characteristics are especially interesting to researchers, such as their high molecular weight [11], the presence of different groups in their composition, and their thermostability and biocompatibility [12]. Another factor to consider is the environment where contamination can occur; many of these environments are characterised by extreme conditions such as elevated or low temperatures, alkaline or acidic pH, high pressure, or high saline concentrations. The bioremediation of these sites is typically difficult [13].

The importance of looking for microorganisms in extreme environments is due to the immense biotechnological potential of their exopolysaccharides (EPSs) [14], since they would be suitable in extreme environments. One of the most versatile genera is *Bacillus,* which is found in different ecological niches [15] and can propagate under adverse conditions [16], rendering the study of its EPSs very interesting [17]. An example is the case of EPSs produced by *Bacillus vallismortis* WF4 [18] and *Bacillus tequilensis* GM [19], which showed significant emulsifying activity in essential oils. The EPS of *Bacillus megaterium* also showed significant emulsifying activity in hydrocarbons [20]. Emulsifying activity was also found for the EPS produced by *Bacillus amyloliquefaciens* [21].

Some EPSs have wide pharmaceutical application. This is the case of *Bacillus thermoantarcticus* [22] and *Bacillus velezensis* [23], which both presented effective antifungal activity, while the EPSs of *Bacillus subtilis* [24] and *Bacillus aerophilus* [25] demonstrated antioxidant activity.

EPSs produced by different strains of the same species have very diverse pharmaceutical, biotechnological, and industrial applications. For example, different strains of *Bacillus licheniformis* have immunomodulatory [26], antiviral [27], and anticytotoxic activity [28].

Another factor to take into account is the culture medium used to stimulate the production of exopolysaccharides. Among the most used synthetic surfactants is Tween 80, composed of polyoxyethylene glycol sorbitan monooleate. This is an important nonionic surfactant, as it is economical and highly efficient [29]. For medical applications, Tween 80 has been recently included in some vaccines, such as the influenza and AstraZeneca COVID-19 vaccines, or as a food additive, and was widely tolerated [30]. While Tween 80 was degraded by bacteria and stimulated the biodegradation process [29], EPSs arising from degrading Tween 80 have not been reported. The hypothesis of this work is that a potential EPS produced by an extremophilic bacterium biodegrading Tween 80 would result in a polymer that could act in a wide pH range.

This study aims to produce a novel EPS_RT7_ from the biodegradation of glucose–Tween 80 with the *Bacillus amyloliquefaciens* RT7 strain, and to understand its potential applications. The extremophilic *Bacillus amyloliquefaciens* RT7 strain was isolated from an extreme acidic environment and identified through molecular biology methods. Furthermore, the biodegradation of the strain using different independent carbon sources (glucose, oleic acid, Tween 80, and PGE 200) and the joint biodegradation of glucose–Tween 80 were evaluated with an indirect impedance technique and nuclear magnetic resonance (NMR). EPS_RT7_ was characterised with different analytical techniques (GC–MS, HPLC/MSMS, ATR–FTIR, TGA and DSC) in order to determine the compositional and structural characteristics, and molecular weight of EPS_RT7_. Lastly, potential applications of the isolated EPS_RT7_, such as emulsifying activity against different natural oils (olive, sunflower, sesame, and coconut) and hydrocarbons (diesel oil, hexane, toluene), the stability of the emulsion at different pH levels, times, and concentrations, and emulsion efficiency against different commercial emulsifiers (Triton X-100, Tween 20 and SDS) were compared. In addition, in the in vitro antioxidant assays for different free radicals, we studied the cytotoxicity and antioxidant activity of EPS_RT7_ at the cellular level.

## 2. Materials and Methods

### 2.1. Chemical and Standards

Olive, sunflower, sesame, and coconut oils (Mercadona, Madrid, MA, ESP). Diesel, hexane, toluene, trypticase soya agar (TSA), dextrans standard, 1,1-diphenyl-2-picryl-hydrazyl radical (DPPH), H_2_O_2_, salicylic acid, Dulbecco’s modified Eagle’s medium (DMEM), polyoxyethylene sorbitan monolaurate (Tween 20), polyoxyethylene glycol sorbitan monooleate (Tween 80), sodium dodecyl sulphate (SDS), 2-[4-(2,4,4-trimethylpentan-2-yl)phenoxy]ethanol (Triton X-100), pyrogallol, HCl, ascorbic acid (Vc), fetal bovine serum (FBS), L-glutamine, penicillin, streptomycin (Sigma-Aldrich, St. Louis, MO, USA). JetQuick kit (Genomed, Leesburg, VA, USA), Sephadex G-100 column (Aldrich Chemical Company, Inc., Milwaukee, WI, USA), trifluoroacetic acid (TFA)(Aldrich^®®^ Schnelldorf, Germany), 3-(4,5-dimethylthiazol-2-yl)-2,5-diphenyltetrazolium bromide (MTM) (GE Healthcare, Uppsala, Sweden). 

### 2.2. Isolation of Bacterial Strain and PCR Amplification 

Extremophilic bacterial strain RT7 was isolated from the sediments of the river source in Río Tinto (Huelva), Spain (37°43′19″ N 6°33′03″ W). First, 10 mL of NaCl 0.6 M was added to 1 g of sediment, and the mixture was serially diluted (10-fold). Aliquots of 100 μL were inoculated on trypticase soya agar (TSA) plates and stored overnight at 30 °C. The isolated strain was preserved at −80 °C in 30% glycerol.

For the identification of the strain, PCR amplification was conducted as described by Abrusci et al. [31]. Genomic DNA was extracted from bacterial cells using an UltraClean microbial DNA isolation kit. The purified genomic DNA was used as a template to amplify the 16S rRNA gene with PCR using primers 27F (5′-AGA GTT TGA TC (C/A) TGG CTC AG-3′) and 1492R (5′-TAC GG(CT) TAC CTT GTTACG ACT T-3′). PCR amplifications were carried out in a Thermal Cycler 2720 (Applied Biosystems). The following were performed: an initial denaturing step at 94 °C for 5 min, the completion of 30 cycles of 1 min at 94 °C, 1 min at 56 °C, and 3 min at 72 °C; and a final extension of 72 °C for 10 min [32]. Amplicons were purified using the JetQuick kit, and sequenced using the ABI PRISM Big Dye Terminator Cycle Sequencing Ready Reaction Kit (ABI) and an Applied Biosystem ABI 310 (PE Applied Biosystems, Foster City, CA, USA) automated sequencer [33]. The obtained sequences were compared to those in the GenBank database using the BLAST program (National Center for Biotechnology Information). The selected sequences were aligned with CLUSTAL X [34].

### 2.3. Biodegradation, Colony-Forming Units (CFUs)/mL, pH, and EPS Production

#### 2.3.1. Biodegradation, Colony-Forming Units (CFUs)/mL, and pH

The biodegrading bacterium (strain RT7) was studied via indirect impedance measurements, performed at 30 °C. The aerobic biodegradation was prepared as previously described by Abrusci et al. [35,36]. Minimal growth medium (MGM): g/L: K_2_HPO_4_ 0.5, KH_2_PO_4_ 0.04, NaCl 0.1, CaCl_2_ 2H_2_O 0.002, (NH_4_)_2_ SO_4_ 0.2, MgSO_4_ 7H_2_O 0.02, FeSO_4_ 0.001. Each of the carbon sources was added separately to the medium (MGM): glucose (4 g/L), oleic acid (1 g/L) and surfactants (1 g/L) (polysorbate 80 (Tween 80), and polyethylene glycol (PEG) 200). Polyethylene glycol PEG-200 and oleic acid were used as controls or model compounds in the biodegradation studies. In addition, glucose and Tween 80 were added together (pH adjusted to 7.0).

The bioassay measurements were performed as described by Abrusci et al. [36] in 7 mL bioreactors, introducing 1.5 mL of bacterial suspension. These bioreactors were introduced into disposable cylindrical cells of 20 mL filled with 1.5 mL of 2 g/L KOH aqueous solution, and impedance was measured using four stainless-steel electrodes on a Bac-Trac 4300 apparatus (SY-LAB Geräte GmbH, Neupurkerdorf, Austria). The typical measurement error was 1–2%. The relative change in the KOH solution impedance value was monitored with the device every 20 min and was converted into carbon dioxide concentration by using a calibration curve of variation in impedance against CO_2_ concentration.

The biodegradation percentage of different carbon sources was computed on the basis of the ratio between the cumulative amount of CO_2_ actually generated by biodegradation at time t and the theoretical carbon dioxide amount, which assumed that all the carbon in the glucose and polysorbate structures was converted into CO_2_ (Formula (1)).

% Biodegradation = ([CO_2_]Prod/[CO_2_]Theor.) × 100 (1)

To continue with the experiments, the most effective carbon source was chosen. The colony-forming units (CFUs) were evaluated via dilution plating incubated at 30 °C for 72 h with a TSA agar medium. A Thermo Orion pH Meter Model 2Star (Thermo Scientific, Asheville, NC, USA) was used to measure the pH values during a 72 h fermentation period.

To evaluate structural changes on Tween 80 after biodegradation, nuclear magnetic resonance (1H-NMR) was recorded in a deuterated chloroform CDCl3 solution on a Varian INOVA-400 instrument (Varian Inc., Palo Alto, CA, USA) at 400 MHz. In the case of the biodegraded products, the compound mixture was filtered to eliminate cells using a centrifuge (0.22 µm Millipore, Merck, Darmstadt, Germany, DEU). The residues were dried and dissolved in CDCl3.

#### 2.3.2. EPS Production and Purification

The strain was inoculated on trypticase soy agar (TSA) medium and incubated for 24 h at 30 °C. The strain was later transferred to an MGM medium in 100 mL flasks filled with 20 mL with an initial inoculum of 2.5 × 10^7^ CFU/mL (OD_550 nm_) measured with a spectrophotometer (Biowave II). The flasks were incubated at 30 °C for 24 h at 110 rpm (Orbitek LJEIL model) [27]. Subsequently, 10 mL of the broth was transferred to flasks containing 1000 mL of MGM with glucose–Tween 80. The flasks were incubated at 30 °C for 72 h at 110 rpm. The tests were independently repeated three times.

The cultures were centrifuged with a DuPont Sorvall RC-5 centrifuge for 30 min at 4 °C at 13.154× *g*. The extracted supernatant was precipitated with three times the volume of ethanol (−80 °C). The EPS was then dialysed for 48 h at 4 °C with Milli-Q water, and lyophilised with a Flexy-Dry MPTM freeze 150 dryer. Subsequently, the dry weight of the EPS was measured.

The purity of the EPS (10 mL, 10 mg/mL) was evaluated with a DEAE-52 anion exchange column (2.6 × 30 cm), and deionised water was used for elution. For this, the used eluents were different concentrations of NaCl (0.2–1.5 M) at a flow rate of 1 mL/min. The phenol–sulfuric acid method was used to monitor the eluents [37]. The collected fractions were lyophilised, resulting in an EPS that was named EPS_RT7_.

### 2.4. Characterisation of EPS_RT7_

#### 2.4.1. Monosaccharide Composition

The molecular weight of EPS_RT7_ was obtained via gel filtration chromatography with a Sephadex G-100 column (1.6 × 50 cm) eluting with 0.2 mol^−1^ NaCl solution at a flow rate of 1 mL/min. Standard reference dextrans (5–80 KDa) were used [38].

The monosaccharide composition was determined with Bruker gas chromatography (EVOC GC–TQ) combined with mass spectrometry using the procedure described by Huang-Lin et al. [39]. For this, EPS_RT7_ hydrolysis was conducted with 0.5 M trifluoroacetic acid (TFA) at 120 °C for 2 h. Subsequently, the samples were treated with N_2_. Galactose, glucose, arabinose, fructose, and xylose were used as the standard. To determine the presence of amino acids and glucuronic acid, an Agilent Technologies 1100 series 6410B (HPLC/MSMS) and an ACE Excel 3 C18-Amide column were used as the stationary phase with a mobile phase of 0.1% formic acid in water. This was conducted at 40 °C (TQ, Waldbronn, Germany) with a flow rate of 0.2 mL/min.

#### 2.4.2. Attenuated Total Reflectance/FT-Infrared Spectroscopy (ATR/FTIR)

The IR spectra of the EPS_RT7_ were recorded using a BX– 187 FTIR spectrometer (Perkin Elmer, Waltham, MA, USA) with an ATR attachment (Pike Technologies, Cottonwood, AZ, USA). The spectra were carried out from accumulating 32 scans at a 4 cm^−1^ resolution over a region from 400 to 4000 cm^−1^ [40].

#### 2.4.3. Thermogravimetric (TGA) and Differential Scanning Calorimetric (DSC) Analysis

TGA was performed with a TGA Q500 (TA Instruments, New Castle, DE, USA). The EPSRT7 (1–3 mg) was placed into a platinum crucible and subjected to temperatures ranging from 20 to 800 °C at a heating rate of 10 °C/min under atmospheric pressure. DSC measurements were conducted using a DSC Q100 (TA Instruments, New Castle, DE, USA). The EPS_RT7_ (0.5–2 mg) was placed in an aluminium pan with its lid removed. The pans were heated from 20 to 600 °C at a rate of 10 °C/min. Data were analysed using TA Universal Analysis software [41].

### 2.5. Emulsifying Activity Assesment

The emulsifying activity of the EPS_RT7_ was measured at different pH levels (7.2, 5.1, 3.1) and at various concentrations (0.5, 1, and 2 mg/mL), using the method described by Meneghine et al. [42]. The experiment involved mixing 1.5 mL of an oil phase (olive oil, sunflower oil, sesame oil, coconut oil, diesel oil, hexane, or toluene) with an aqueous phase of 1.5 mL. For the aqueous phase, the commercial emulsifiers of Tween 20, sodium dodecyl sulphate (SDS), Triton X-100, and EPS_RT7_ were compared. The tubes were stirred for 2 min at 2400 rpm using a vortex. Emulsification indices E24, E48, and E168 were measured after 24, 48, and 168 h, respectively. Formula (2) was used to calculate the emulsification indices:

E [%] = HEL/HT × 100(2)

where HEL (mm) represents the height of the emulsion layer, and HT (mm) refers to the total height.

### 2.6. Antioxidant Activity Assesments 

To evaluate the antioxidant properties of EPS_RT7_, several tests were conducted using 1,1-diphenyl-2-picryl-hydrazyl radical (DPPH•), hydroxyl radical (•OH), and superoxide anion (O_2_^−^•) as indicators. Ascorbic acid (Vc) was used as the positive control. EPS_RT7_ was prepared in concentrations ranging from 0.1 to 10 mg/mL. Absorbance measurements were performed using a FLUOstar Omega BMG LABTECH (Aylesbury, UK) spectrophotometer for DPPH (OD_525 nm_), OH (OD_510 nm_), and O_2_^−^(OD_325 nm_). 

#### 2.6.1. DPPH Radical Scavenging Activity

Research on the DPPH scavenging activity of EPS_RT7_ followed the procedure described by Niknezhad et al. [43]. First, 50 μL of EPS_RT7_ at different concentrations was mixed with 100 μL of DPPH (100 μM DPPH–ethanolic solution). The mixtures were stirred and left to incubate in the dark at 25 °C. The absorbance was measured after 30 min.

Formula (3) was used to determine the percentage of radical-scavenging activity for DPPH.

DPPH scavenging activity [%] = [ 1 − (A_1_ − A_2_)/A_0_] × 100 (3)

where A_1_ represents the reaction mixture, A_2_ refers to the reaction mixture without DPPH, and A_0_ denotes the reaction mixture with DPPH and without EPS_RT7_.

#### 2.6.2. OH Radical Scavenging Activity 

The FeSO_4_–salicylic acid method described by Sun et al. [44] was used to determine the hydroxyl radical scavenging activity of EPS_RT7_. The mixtures contained a FeSO_4_ solution (9 mM, 40 µL), 40 µL of a 9 mM ethanol–salicylic acid solution, EPS_RT7_ diluted at various concentrations (40 µL), and H_2_O_2_ (8.8 mM, 40 µL). The mixtures had then been incubated at 37 °C for 30 min before absorbance was measured. Formula (4) was used to determine the percentage of hydroxyl radical scavenging activity.

Hydroxyl radical scavenging activity [%] = [ 1 − (A_1_ − A_2_)/A_0_] × 100 (4)

where A_1_ represents the reaction mixture, A_2_ refers to the reaction mixture without salicylic acid, and A_0_ denotes the reaction mixture with salicylic acid and without EPS_RT7_.

#### 2.6.3. O_2_^−^ Scavenging Activity 

The superoxide scavenging activity of EPS_RT7_ was assessed as per Balakrishnan et al. [45]. In this method, 0.3 mL of different EPS_RT7_ concentrations was mixed with 2.6 mL of phosphate buffer (50 mM, pH 8.2) and 90 μL of pyrogallol (3 mM), and dissolved in HCl (10 mM). The absorbance was then monitored at 0 and 10 min. Formula (5) was used to determine the percentage of superoxide scavenging activity:

Superoxide scavenging activity [%] = 1 − [(A_10_/C_10_) − (A_0_/C_0_)] × 100 (5)

where A_0_ and A_10_ represent the reaction mixture at 0 and 10 min, respectively; C_0_ and C_10_ represent the reaction mixture without pyrogallol at 0 and 10 min, respectively.

### 2.7. Exopolysaccharide Toxicity Evaluation 

#### 2.7.1. Cell Culture

HeLa cells, which are human epithelial cells derived from cervical carcinoma, were obtained from CLS (Cell Line 76 Service, BW, Eppelheim, Germany) and chosen as the reference cell line to assess the toxicity of EPS_RT7_. The cells were cultured using Dulbecco’s Modified Eagle’s Medium (DMEM) and added to 10% fetal bovine serum (FBS), 2 mM L-glutamine, penicillin (100 IU/mL), and streptomycin (100 μg/mL) under a 5% CO_2_ atmosphere at 37 °C [46].

#### 2.7.2. Cytotoxicity Assay 

HeLa cells were cultured in a 24-well culture plate at a density of 5 × 10^5^ cells per well. Each well was treated with 100 µL of EPS_RT7_ at various concentrations (0–400 µg/mL) for 24 h. The toxicity of the exopolysaccharide was determined by measuring the reduction in the MTT reagent (3-[4,5-dimethyl-thiazol-2-yl]-2,5-diphenyltetrazoliumbromide) to formazan [46,47]. The optical density at a wavelength of 590 nm was recorded using a microplate reader.

The cytotoxicity of EPS_RT7_ on HeLa cells was assessed using Formula (6):

Cell viability [%] = (A_1_/A_2_) × 100 (6)

where A_1_ refers to cells treated with EPS_RT7_ and the MTT solution, and A_2_ refers to cells without any treatment with the MTT solution.

### 2.8. Evaluation of Antioxidant Ability on HeLa Cells 

#### 2.8.1. Injury Inducement Model 

The methodology used to create an injury model for HeLa cells was based on the procedure outlined by Huang-Lin et al. [39]. HeLa cells were seeded at a density of 5 × 10^4^ cells per well for 24 h. The medum was then removed and replaced with 100 µL of various concentrations of H_2_O_2_ (0.25–2 mM) for 1 h at 37 °C under a 5% CO_2_ atmosphere. Following exposure, the H_2_O_2_ solution was removed, and a fresh medium was added to the wells. Cell viability was measured using the MTT method as described in Section 2.7.2.

HeLa cell viability was computed using Formula (7):

Cell viability (%) = (A_1_/A_2_) × 100 (7)

where A_1_ represents the absorbance of HeLa cells treated with H_2_O_2_ and the MTT solution, while A_2_ represents the absorbance of HeLa cells that were not subjected to any treatment with the MTT solution.

#### 2.8.2. Evaluation of Protective EPS_RT7_ Effect on HeLa Cells from Oxidative Stress 

EPS_RT7′_s ability to protect HeLa cells against oxidative stress was evaluated following the methods described by Huang-Lin et al. [39]. HeLa cells were seeded at a concentration of 5 × 10^4^ cell/well and incubated for 24 h. After that, the DMEM solutions were withdrawn and substituted with EPS_RT7_ diluted in DMEM at different concentrations (25–400 µg/mL). After 1 h, the EPS_RT7_ solutions were removed, and a new medium containing 2 mM of H_2_O_2_ was added and incubated for 1 h more. The MTT method described in Section 2.7.2 was used to determine cell viability. As a positive control, ascorbic acid (20 mg/mL) was used.

HeLa cell viability was calculated with Formula (8):

Cell viability [%] = (A_1_/A_2_) × 100 (8)

where A_1_ refers to cells that were treated with both H_2_O_2_ and EPS_RT7_, and subsequently exposed to the MTT solution; A_2_ refers to cells that did not receive any treatment and were exposed to the MTT solution.

### 2.9. Statistical Analysis

The experiments were conducted three times, and statistical analysis was performed using the Statistical Package for the Social Sciences (SPSS), version 21. The analysis of variance (ANOVA) test was used for statistical comparison, and statistical significance was at *p* < 0.05.

## 3. Results and Discussion

### 3.1. Bacterial Identification, Biodegradation, and EPS Production

The bacterial strain was isolated as described in Section 2.2, and identified after PCR amplification and sequencing using the 16S rDNA sequence. The 16S rDNA sequences were compared with those in the GenBank database and showed that the isolated strain was *B. amyloliquefaciens* RT7 (accession number, AB300821) with a similarity of 98%.

*Bacillus amyloliquefaciens* RT7 is a Gram-positive, endospore-forming bacterium isolated from the sediments of Rio Tinto (Huelva, Spain), which is one of the most acidic rock drainage fluvial–estuarine systems in the world [48]. This species is ubiquitous and adapts to very different ecological environments. This gives it great versatility in the biodegradation of different compounds, such as waste from the petrochemical industry, where strain *B. amyloliquefaciens* W1 [49] was able to degrade benzene, toluene, ethylbenzene, and xylene (BTEX), also in phenol-contaminated wastewater, which *B. amyloliquefaciens* WJDB-1 was able to biodegrade [50]. In addition, it was successfully used in various technological applications. For example, the *B. amyloliquefaciens* BRRI53 strain could stimulate plant growth [51], and the *B. amyloliquefaciens* BPRGS strain had high flocculant activity [52].

**Figure 1:**
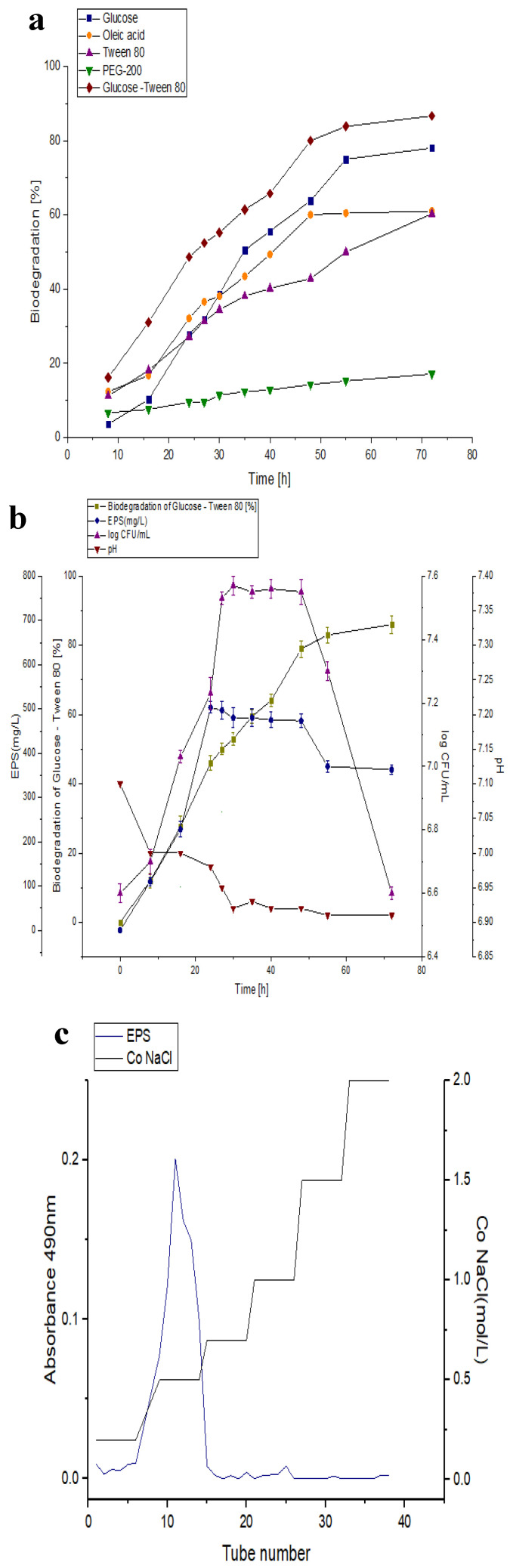
(**a**) Biodegradation study of different carbon sources (glucose, oleic acid, Tween 80, PEG 200 and glucose–Tween 80) from *Bacillus amyloliquefaciens*. (**b**) Optimisation of the production EPS from *B. amyloliquefaciens*. (**c**) Elution curve obtained from the purification of EPS_RT7_ under MGM with glucose–Tween 80.

The biodegradation of the different independent carbon sources (glucose, oleic acid, Tween 80, and PEG 200) and the joint biodegradation of glucose–Tween 80 are shown in Figure 1a. The results indicate that the *B. amyloliquefaciens* RT7 strain biodegraded glucose by 27–78%, oleic acid by 27–60%, Tween 80 by 23–58%, PEG-200 by 5–2%, and the combination of glucose–Tween 80 by 46–86% at intervals of 24 and 72 h. The *B. amyloliquefaciens* RT7 strain was the most effective at biodegrading glucose–Tween 80.

The growth of *B. amyloliquefaciens* RT7, pH values, medium biodegradation, and exopolymer production (EPS) at 30 °C using the combination of glucose–Tween 80 as a carbon source is shown in Figure 1b. Cell growth peaked (7.53 log CFU/mL) after 30 h. During the process, the medium was not acutely acidified (from pH 7 to pH 6.5).

**Figure 2:**
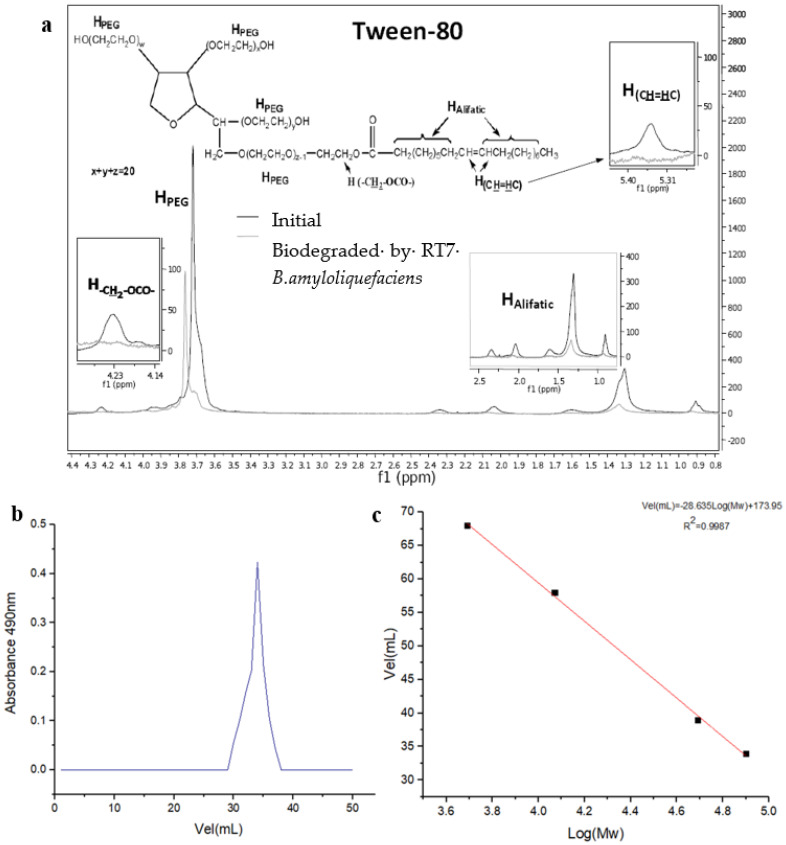
(**a**) The 1H-NMR spectra of Tween 80 initially and after 72 h of biodegradation. (**b**) The elution curve of EPS_RT7_ by Sephadex G-100 gel filtration. (**c**) The standard curve of relative molecular weight (Mw).

To confirm that Tween 80 had undergone structural changes during biodegradation, after the bioassays, the residues were analysed with proton nuclear magnetic resonance (1H-NMR; Figure 2a). The biodegradation of oleic segments decreased the intensity of the peaks of the aliphatic protons (2.5–1.0 ppm), and caused the disappearance of the double-bond signal at 5.35 ppm and the methylene protons next to the oleic ester group at 4.23 ppm. We also found a simplification of the proton signals corresponding to the PEG fragments (HPEG–3.64, 3.77, and 3.99–4.16 ppm), indicating a decrease in the length of the initial PEG fragments after 72 h of the bioassay. This shows that the biodegradation of Tween 80 [53] was effective and likely had a positive effect on the biodegradation of glucose. Similar results were obtained with *Bacillus amyloliquefaciens* [54] when Tween 80 was combined with hydrocarbons, favouring the biodegradation of the latter. This was also confirmed in other species of the *Bacillus* genus, such as *B. subtilis* ZL09-26, where the biodegradation of phenanthrene was more efficient in the presence of Tween 80 [55]. This could have been due to the fact that Tween 80 could influence the permeability of the membrane, improving the expression of the proteins [56] and favouring the more efficient incorporation of organic nutrients such as glucose [57,58].

On the other hand, the biodegradation of this combination (glucose–Tween 80; Figure 1b) with *Bacillus amyloliquefaciens* RT7 resulted in a high production of EPS. The maximal production of the exopolysaccharide, namely, 490 mg/L, occurred at 24 h, during the exponential growth phase. However, this was previously observed as usually taking place at the beginning of the stationary phase [27,39].

The EPS production of the RT7 strain was higher than that for other strains of *B. amyloliquefaciens*. *B. amyloliquefaciens* p16 used a nutrient broth of glucose, peptone, and yeast as an energy source, and only had an EPS production of 223.87 mg/L [59]. The use of Tween 80 by *Bacillus amyloliquefaciens* RT7 as a carbon source not only allowed for greater efficiency in the use of glucose, but also effectively accelerated the synthesis of the exopolysaccharide produced by the RT7 strain. Similar processes were described in the synthesis of natural compounds, such as fengycin, accelerating their production when Tween 80 was used [60]. In addition to this, similar results were found in other genera, such as *Lactobacillus plantarum*, where Tween 80 not only facilitated the entry of nutrients, but also stimulated greater EPS production [61].

The EPS obtained from glucose and Tween 80 as an energy source was purified and showed a single characteristic peak of exopolysaccharides with high purity (Figure 2b). The result of the obtained fraction from the purified exopolymer was named EPS_RT7_. The estimated molecular weight of EPS_RT7_ was about 7.0794 × 10^4^ Da (Figure 2c), which fell within the typical molecular weight range for heteropolysaccharides (4 × 10^4^ and 6 × 10^6^ Da) [62]. EPS_RT7_ presented a high molecular weight in comparison with that of other EPS produced without the presence of Tween 80, such as strains of *B. amyloliquefaciens* GSBa-1, with an EPS composed of glucose with a molecular weight of 5.4 × 10^4^ Da [63], and *B. amyloliquefaciens* 3MS [64], whose EPS comprised glucose, galactose, and glucuronic acid with a molecular mass of 3.76 × 10^4^ Da.

### 3.2. EPS_RT7_ Compositional Analysis and Characterisation 

#### 3.2.1. GC–MS Analysis for EPS_RT7_

**Figure 3:**
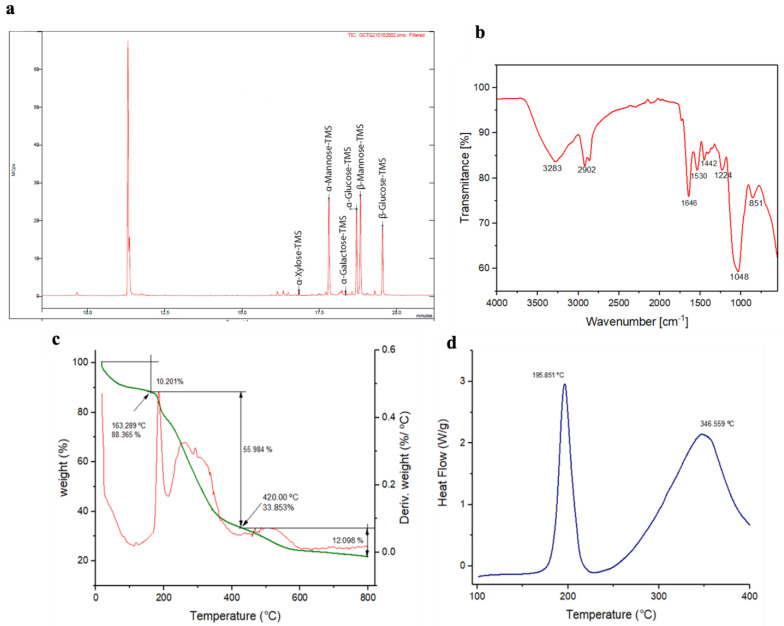
Characterisation analysis of EPS_RT7_. (**a**) GC–MS; (**b**) ATR–FTIR spectra; (**c**) thermogravimetric analysis (TGA); (**d**) differential scanning calorimetry (DSC).

Gas chromatography (GC–MS) analysis (Figure 3a) exhibited 4 peaks that corresponded to monosaccharides mannose (α-D-mannose and β-D-mannose), glucose (α-D-glucose and β-D-glucose), α-D-galactose, α-D-xylose, with a molar ratio of 1:0.5:0.1:0, respectively. This revealed that the polymer was a heteropolysaccharide with the absence of uronic acids and amino acids. *B. amyloliquefaciens* strains produce a wide variety of different EPS compositions. *B. amyloliquefaciens* strain JN4 [65] produced an EPS that was composed of fructose and glucose with a molar ratio of 46.1:1. *B. amyloliquefaciens* strain C-1 [66] produced two different EPSs: EPS-1 with glucose, mannose, galactose, and arabinose at a 15:4:2:1 ratio, and EPS-2, composed of glucose/mannose at a 3:1 proportion. On the other hand, heteropolysaccharide EPS-K4, isolated from the *B. amyloliquefaciens* DMBA- K4 strain, contained rhamnose, mannose, glucose, and glucuronic acid at a molar ratio of 23.65:40.09:17.68:11.42 [67]. 

#### 3.2.2. ATR–FTIR Analysis for EPS_RT7_

In addition, ATR–FTIR spectra (Figure 3b) obtained from EPS_RT7_ produced with *B. amyloliquefaciens* RT7 showed peaks between 4000 and 400 cm^−1^, a characteristic of carbohydrates. A band at 3283 cm^−1^ was attributed to the hydroxyl stretching vibration of the polysaccharide [68], whereas the band at 2902 cm^−1^ was ascribed to C–H stretching vibration [69]. The peaks at 1664 and 1442 cm^−1^ were attributed to the stretching vibration of C=O, which is characteristic of a carboxyl group [70,71]. The peak at 1530 cm^−1^ was ascribed to C–O vibration [72]. The region at 950–1250 cm^−1^ corresponded to CO and C–O–C stretching vibrations in carbohydrates. The absorption at 1224 cm^−1^ could have been the pyranose ring of monosaccharides in EPS_RT7_. The strong peak at 1048 cm^−1^ was attributed to the C–O–C glycosidic bond vibration, which indicated the pyranose configuration [73]. The band at 851 cm^−1^ suggested that the EPS_RT7_ contained the α and ß configurations of the glucose unit [74].

The spectrum confirmed typical characteristics of a polysaccharide. The vibrational peaks resembled the peaks observed for the EPSs of other strains. In the case of EPS from *B. amyloliquefaciens* RK3, the polymer contained mannose and galactose [75]. These differences revealed that the exopolysaccharides of the different strains of *B. amyloliquefaciens* had a very varied composition of monosaccharides. This heterogeneity may have been due to both nutritional and environmental factors.

#### 3.2.3. Characterisation of the Thermal Properties of EPS_RT7_

The thermal stability of EPSs is an important characteristic for their commercial utilisation. The thermal decomposition curve (TGA) of EPS_RT7_ is shown in Figure 3c. The first step, with initial weight loss of 10.20%, was observed at around 25 and 163 °C, mainly due to the moisture loss in EPS_RT7_. The second step had a weight loss of approximately 55.9% which reached the maximum at around 420 °C. The depolymerisation of the polysaccharide and the thermal chemical-bond scission occurred, accompanied by the dehydration of sugar units. Lastly, the EPS presented a gradual weight loss of approximately 12.09%, reaching equilibrium with only 28% of the remaining residue. Similar results associated with thermal decomposition were found in other strains of *B. amyloliquefaciens*. This was the case of the *B. amyloliquefaciens* GSBa-1 strain [63], whose EPS was formed by glucose. In the first step, 7.44% (50 to 160 °C) weight loss for water was observed. In the second step, it was 43% (around 400 °C) of mass loss. In *B. amyloliquefaciens* BPRGS [52], the first step showed 10% weight loss for water (0 to 180 °C), and the second step showed 20.21% weight loss (250 to 550 °C). On the other hand, the DSC thermogram of EPS_RT7_ (Figure 3d) showed two melting peaks at 195.8 and 346.5 °C that conferred higher thermostability. The appearance of two melting peaks was directly related to the heterogeneity in the composition of EPS_RT7_ sugars. This was in contrast with the case of *B. amyloliquefaciens* LPL061 [21]. This strain produced two distinct EPSs, both composed of mannose and glucose (EPS1 and EPS2). The two EPSs had a single melting peak of 224.09 °C (EPS1) and 301.09 °C (EPS2). This suggests that compositions with more distinct sugars may result in more than one melting points. Therefore, EPS_RT7_ with various sugars presented an important advantage over polymers with fewer sugars, since its thermostability increased, which is an important factor in various industrial applications [21,76].

### 3.3. Biotechnological Applications

#### 3.3.1. Emulsifying Activity

**Figure 4:**
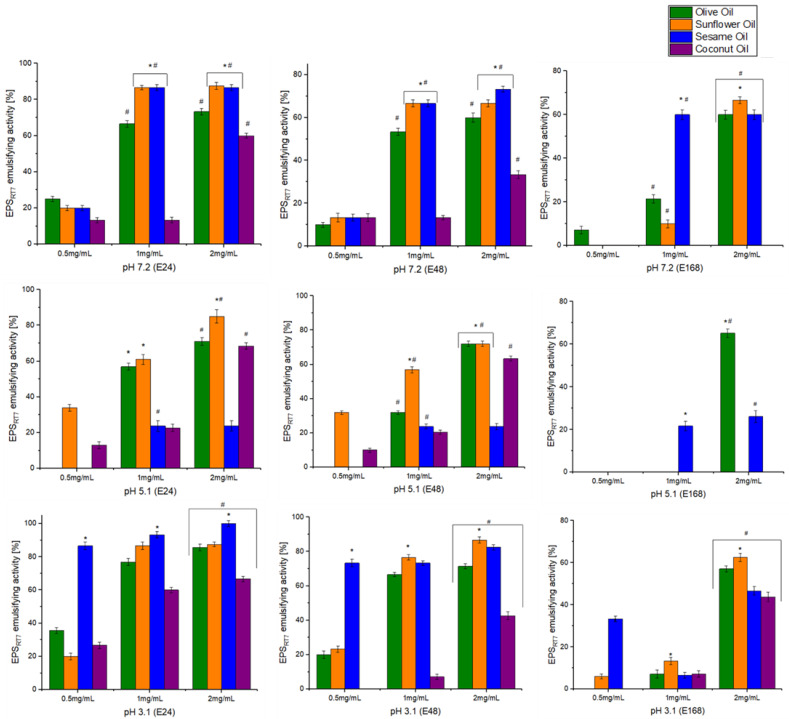
Emulsifying activity for natural oils with different EPS_RT7_ concentrations (0.5, 1, 2 mg/mL) and pH levels (7.2, 5.1, 3.1). Emulsion percentages of EPS_RT7_ with different oils used at 24, 48, and 168 h of study are also shown. *, statistical differences between oils for each concentration (*p* < 0.05). #, statistical differences between concentrations (*p* < 0.05).

**Figure 5:**
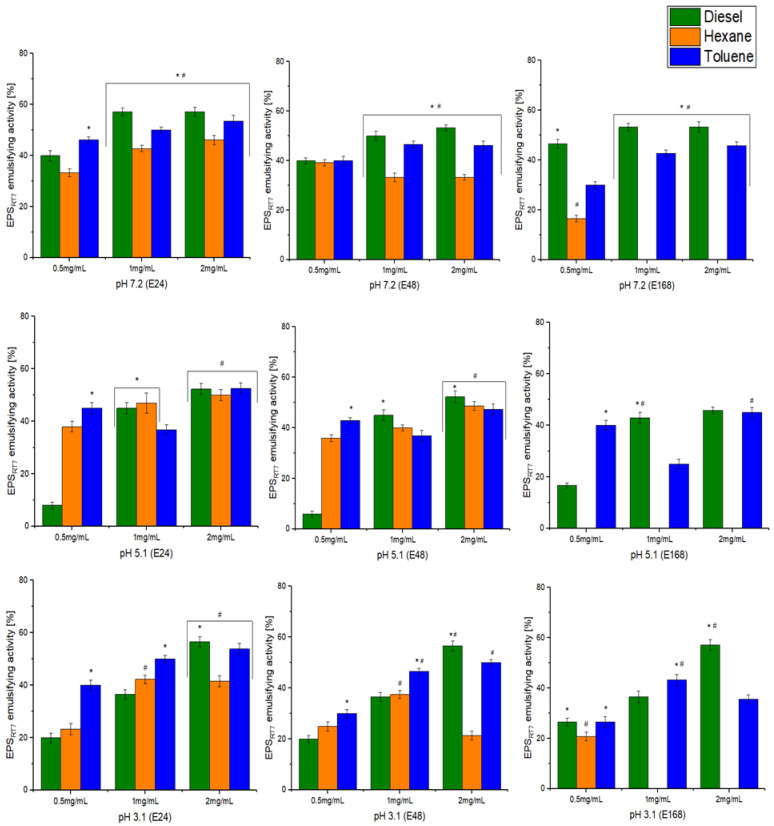
Emulsifying activity for hydrocarbons with different EPS_RT7_ concentrations (0.5, 1, 2 mg/mL) and pH levels (7.2, 5.1, 3.1), measured at 24, 48, and 168 h of study. *, statistical differences between different hydrocarbons for each concentration (*p* < 0.05). #, statistical differences between different concentrations (*p* < 0.05).

The emulsification behaviour of EPS_RT7_ was investigated at different concentrations (0.5, 1, and 2 mg/mL), pH levels (7.2, 5.1, 3.1), and at different times (24 h (E24), 48 h (E48), and 168 h (E168)), with natural oils (olive, sunflower, sesame, and coconut) and hydrocarbons (diesel oil, hexane, toluene) (Figure 4 and Figure 5). EPS_RT7_ was also tested with three commercial emulsifiers (Triton X-100, Tween 20, and SDS). 

For natural oils, concentration has a statistically significant effect on emulsifying activity. Concentrations of 0.5 mg/mL resulted in nonsignificant emulsification activity across the board, with the only exception being sesame oil at pH 3.1 ((E24 86.6%), (E48 73.3%)). A concentration of 1 mg/mL generally resulted in emulsifying activity with some exceptions, but the optimal concentration was 2 mg/mL for all oils and pH and time combinations. pH had different effects on emulsifying activity depending on the oil. For olive oil (2 mg/mL), the exopolysaccharide presented significant emulsifying activity at all pH levels and studied times, with pH 3.1 being where the emulsifying activity reached its maximum (E24 (85.7%), E48 (71.4%), E168 (57.1%)). For sunflower oil (2 mg/mL), there was significant emulsifying activity for all pH levels; however, at pH 5.1, emulsification was not maintained at E168 ((E24 (85%), E48 (72%), E168 (Nd)). Sunflower oil had its highest emulsifying activity at pH 3.1 (E24 (87.5%), E48 (86.6%), E168 (62.5%)).

The EPS was initially most effective in emulsifying sesame oil at pH 3.1, although this was not maintained at 168 h (E24 (100%), E48 (82.4%), E168 (46.6%)). However, at pH 7.2, emulsification activity was maintained (E24 (86.6%), E48 (73.3%), E168 (60%)), and it was not successful at a pH 5.1. For coconut oil, emulsifying activity was similar at different pH levels for E24, but for E48, pH 5.1 was most effective, followed by pH 3.1; for E168, only pH 3.1 still presented some emulsification. Similar results were obtained with the EPS of the *B. amyloliquefacens* ZWJ strain [77], where the optimal concentration was 1.5 mg/L for two natural oils (olive oil (96.2%), sunflower oil (76%)). In the case of *B. amyloliquefaciens* LPL061 [21], the emulsifying activity was lower than that presented by EPS_RT7_. Its EPS was only tested at a concentration of 1 mg/mL. The EPS of this strain showed emulsifying activity with the natural oils, which did not exceed 66% (olive oil (58.6%), sunflower oil (65.8%), peanut oil (60.3%), rice oil (58.5%)).

Similarly, for hydrocarbons, a concentration of 2 mg/mL was significantly the most effective for emulsification. Diesel, (pH 7.2 (E24 (57.1%), E48 (53.3%), E168 (53.3%)) and pH 3.1, (E24 (56.6%), E48 (56.6%), E168 (57.1%)), and toluene (pH 3.1 (E24 (53.8%) E48 (50%)) presented the highest emulsification activity, which was maintained across different pH levels. Hexane only presented emulsification activity of over 50 for 2 mg/mL and pH 5.1, measured at E24. There are few studies that found EPSs produced by other strains of *B. amyloliquefaciens* with the ability to emulsify hydrocarbons. The An6 strain of *B. amyloliquefaciens* [78] produced a biosurfactant that had emulsifying activity of 80% with diesel at a pH range of 5.0–9.0, but this biosurfactant was not confirmed as an EPS, as its chemical composition was inconclusive. This ability has previously been found in other genera. *Bacillus subtilis* AF17 [79] produced an EPS that was capable of emulsifying diesel by 17%, hexane by 72%, and toluene by 84% (5 mg/mL). The stabilisation of the EPS emulsions was specific for certain hydrophobic compounds [80,81].

**Figure 6:**
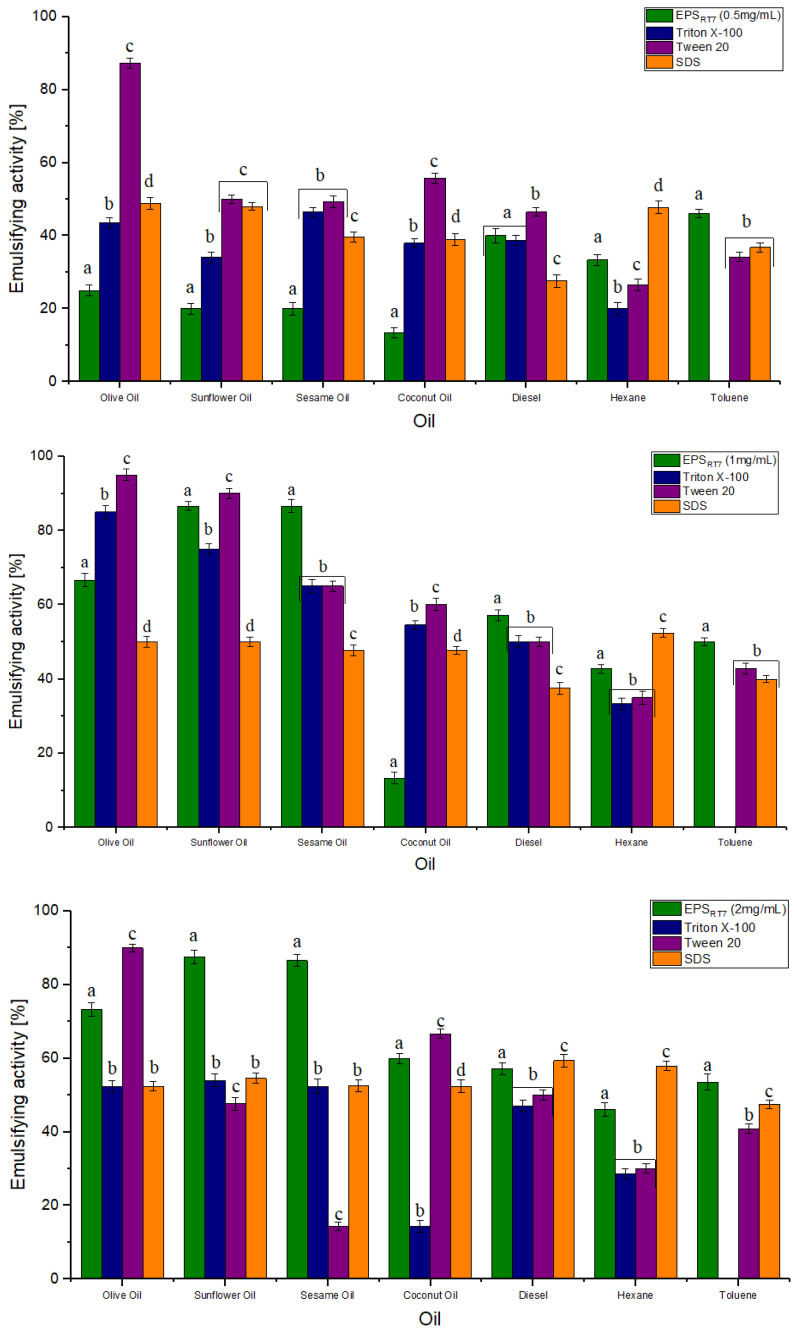
Comparison of emulsifying activity at different EPS_RT7_ concentrations (0.5, 1, 2 mg/mL) against commercial emulsifiers (Triton X-100, Tween 20 and SDS) across different natural oils and hydrocarbons. Different letters (a–d) represent the statistical difference between different emulsifiers for each natural oils and hydrocarbons (*p* < 0.05).

The EPS was compared to commercial emulsifiers (Triton X-100, Tween 20 and SDS) for both natural oils and hydrocarbons, and had similar emulsifying activity to theirs at pH 7.2 (Figure 6). At a concentration of 0.5 mg/mL, both EPS_RT7_ and commercial emulsifiers were not effective (below 50%) except for Tween 20 for olive oil (E24 87.3%), sunflower oil (E24 50.0%), and coconut oil (E24 55.8%). For the concentration of 1 mg/mL, EPS_RT7_ presented significantly higher emulsification than that of SDS, except for coconut oil (E24 47.62%) and hexane (E24 52.38%), higher than that of Triton X-100 except for olive oil (E24 86%) and coconut oil (E24 54.5%), and higher than that of Tween 20 except for olive oil (E24 95%), sunflower oil (E24 90%), and coconut oil (E24 60%). For the concentration of 2 mg/mL, EPS_RT7_ presented significantly higher emulsification than that of SDS except for diesel (E24 59.38%) and hexane (E24 57.89%), higherthan that of Triton X-100 for all natural oils and hydrocarbons, and higher than that of Tween 20 except for olive oil (E24 90%) and coconut oil (E24 66.7%).

The molecular compositions, molecular weight, and functional groups of EPSs have important effects on emulsification. EPS_RT7_ had good emulsifying activity at high concentrations and the studied pH range, which may be attributed to electrostatic interaction and interactions between hydrophilic groups [82]. A low pH, such as 3.1, has a negative effect on the emulsification activity of commercial polysaccharides such as xanthan and Arabic gum [43]. However, in this case, EPS_RT7_ emulsifying activity was not affected at pH 3.1. The capability of EPS_RT7_ to emulsify at different pH levels and thus its ability to bioremediate different environments, and its nontoxicity give it great advantages over other EPSs and commercial emulsifiers.

#### 3.3.2. Antioxidant Effect

The EPS_RT7_ obtained from the biodegradation of the combination of glucose and Tween 80 with *B. amyloquefaciens* RT7 was tested in order to study its potential and benefits, particularly antioxidant and emulsifying activities. To quantify antioxidant activity, the method described in Section 2.6 was used.

**Figure 7:**
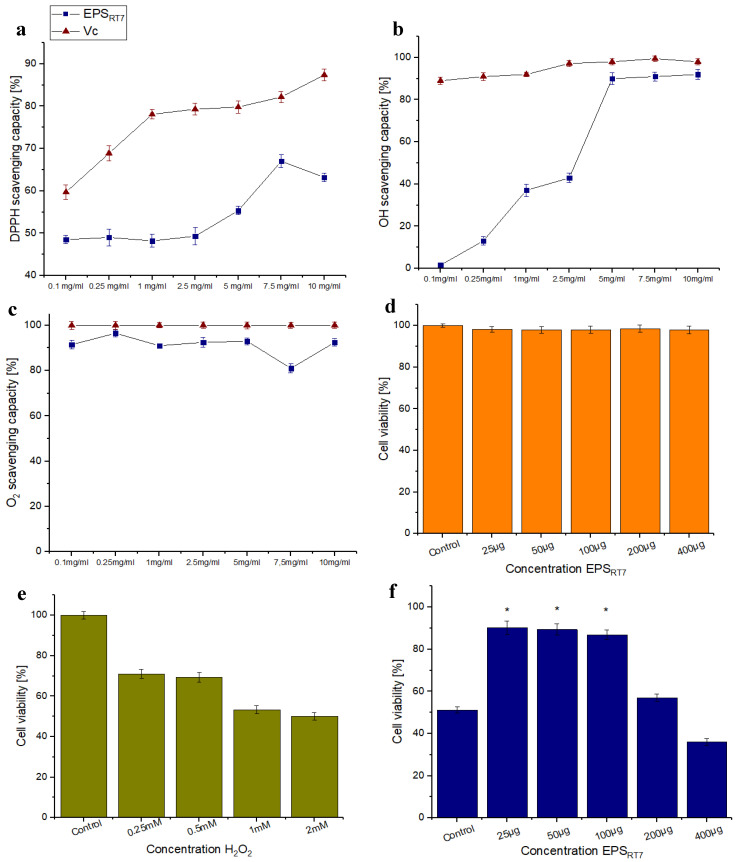
Antioxidant tests and toxicity evaluation with different concentrations of EPS_RT7_. (**a**) DPPH free radical scavenging activity. (**b**) Hydroxyl radical scavenging activity of EPS_RT7_ (**c**) Superoxide anion scavenging activity. (**d**) Hela cells viability (%) by different EPS_RT7_ concentrations. (**e**) Hela cells viability (%) against oxidative stress by different H_2_O_2_ concentrations. (**f**) Exhibition of EPS_RT7_ protection on Hela cells viability (%). (* *p* < 0.05).

DPPH evaluates the radical scavenging activity of nonenzymatic antioxidants. As shown in Figure 7a, the scavenging activity of EPS_RT7_ on DPPH radicals did not increase in a concentration-dependent manner. EPS_RT7_ presented its highest scavenging activity of 67% at 7.5 mg/mL, while for the same concentration, the scavenging capability of Vc was 82%. This was close to the one previously presented by the *B. amyloliquefaciens* GSBa-1 strain [63], where the EPS had a DPPH scavenging activity of 76.7% (5 mg/mL/Vc 90%). The potential of EPS_RT7_ for DPPH inhibition suggests that it had enough proton donors to convert free radicals into stable molecules [83].

The hydroxyl radical is one of the most reactive free radicals in a biological system [84]. This is a type of free radical with the most active chemical properties and that can cause the most harm in comparison to other free radicals, as it damages DNA base sequences [85]. The scavenging activity of the hydroxyl radical is commonly used to evaluate the ability of compounds to scavenge free radicals. Figure 7b shows the results for hydroxyl radical scavenging activity of the EPS_RT7_. Scavenging activity increased in proportion to the concentration. At a concentration of 5 mg/mL, the scavenging capability of EPS_RT7_ was 90% and remained constant, whereas the activity of Vc was 98% at the same concentration. This efficacy is similar to that previously obtained by the *B. amyloliquefaciens* GSBa-1 strain: 89.7% (5 mg/mL/Vc 90%) [63]. However, the EPSs obtained from *B. amyloliquefaciens* C-1 [66] were much less efficient than EPS_RT7_, since they presented hydroxyl radical activity of 60.4% for EPS-1, and less than 10% for EPS-2 at 5 mg/mL (Vc 100%). The high efficiency of EPS_RT7_ could have been due to the bond dissociation energy of EPS_RT7_ being relatively weak; therefore, it was easy to provide more electron atoms to bind to the hydroxyl radical [86].

Superoxide radicals can be harmful to cells, and their anions can increment damage to cellular components, as they generate oxidising agents and other free radicals. Figure 7c shows the superoxide anion scavenging activity of EPS_RT7_. At the concentration of 0.25 mg/mL, the scavenging capability of EPS_RT7_ was very high, 96.5% (Vc 100%). The superoxidant anion scavenging of EPS_RT7_ was higher than the previously studied EPS of *B. amyloliquefaciens* GSBa-1 [63] of with 44.8% (5 mg/mL/Vc 99.1%), and both EPS1 and EPS2 produced by the *B. amyloliquefaciens* C1 strain (EPS1: 30.8% (5 mg/mL/Vc 99.1%), EPS2: 8.5% (5 mg/mL/Vc 99.1%)) [66]. The superoxide anion scavenging mechanism was associated with O–H bond dissociation energy [75].

#### 3.3.3. Toxicity Evaluation and Antioxidant Ability at the Cellular Level

Figure 7 shows the biocompatibility and antioxidant capacity of EPS_RT7_ at the cellular level. For all tested polymer concentrations (Figure 7d), HeLa cells did not show statistically significant damage (*p* < 0.05). This implies that EPS_RT7_ did not cause cytotoxicity in the cell line. Similar results were reported for *B. amyloliquefaciens* amy-1, where cytotoxicity assays revealed that EPSs at 50–800 μg/mL were not toxic to the human enteroendocrine cell line, NCI-H716 cells [87]. On the other hand, other biocompatibility studies for the same species revealed that, for concentrations in the range of 200–800 μg/mL of EPS, HEK293T cells moderately inhibited their growth [88].

Figure 7e shows the cell viability of HeLa cells after being treated to different concentrations of H_2_O_2_. Lower cell viability of below 70% was detected due to oxidative stress in the cells resulting from a build-up of reactive oxygen species (ROSs). The antioxidant effect of EPS_RT7_ on the cell line (Figure 7f) was tested at different concentrations. A statistically significant increase in cell viability was observed in concentrations between 25 and 100 µg/mL. The results demonstrate that EPS_RT7_ concentrations in the range of 25–100 µg/mL statistically significantly improved cellular viability. These results indicate that EPS_RT7_ significantly protected HeLa cells from H_2_O_2_-induced cytotoxicity. Cells incubated with 25, 50, and 100 µg/mL EPS_RT7_ grew better (90.2, 89.4, 86.9%, respectively) than cells of the control group did, indicating that EPS_RT7_ had a growth-promoting effect on normal cells. Similar results were reported with the pretreatment of EPS isolated from *Bacillus amyloliquefaciens* significantly and time-dependently decreasing the ROSs induced by H_2_O_2_ for H_2_O_2_-treated HepG2 cells [66]. These results suggest that EPS_RT7_ from *Bacillus amyloliquefaciens* could promote the antioxidant system by stimulating enzymes to have this capacity [89].

## 4. Conclusions

*Bacillus amyloliquefaciens* RT7 was isolated from the sediments of Rio Tinto. Biodegradation was effective for the different independent carbon sources (glucose, oleic acid, Tween 80, and PEG 200) and the joint biodegradation of glucose–Tween 80. The latter was the most effective, where high EPS production occurred—490 mg/L at 24 h. Polymer characterisation identified the extracted EPS as a heteropolysaccharide composed of mannose, glucose, galactose, and xylose (molar ratio 1:0.5:0.1:0.1). O–H, C=O, and C–O groups were detected within EPS_RT7_ using structural analysis. EPS_RT7_ had an approximate molecular weight of 7.0794 × 10^4^ Da with good thermostability. The EPS also showed emulsifying activity against some natural oils (olive, sunflower, sesame, and coconut) and hydrocarbons (diesel oil, hexane, toluene) when used at a 2 mg/mL concentration and the studied pH range, thus demonstrating its ability to bioremediate different environments. EPS_RT7_ demonstrated its potential as an antioxidant during in vitro antioxidant assays, as it showed robust radical scavenging activity. It was also nontoxic and showed cellular biocompatibility while providing protection to cells damaged by ROSs. The strong emulsifying activity coupled with its antioxidant effect and lack of cytotoxicity suggest it could have promising applications in bioremediation processes, and offers great advantages over other EPSs and commercial emulsifiers.

## Data Availability

Data sharing is not available for this article.
